# Loss of Integrity of Corpus Callosum White Matter Hyperintensity Penumbra Predicts Cognitive Decline in Patients With Subcortical Vascular Mild Cognitive Impairment

**DOI:** 10.3389/fnagi.2021.605900

**Published:** 2021-02-18

**Authors:** Yage Qiu, Ling Yu, Xin Ge, Yawen Sun, Yao Wang, Xiaowei Wu, Qun Xu, Yan Zhou, Jianrong Xu

**Affiliations:** ^1^Department of Radiology, Renji Hospital, School of Medicine, Shanghai Jiao Tong University, Shanghai, China; ^2^Department of Neurology, Renji Hospital, School of Medicine, Shanghai Jiao Tong University, Shanghai, China; ^3^Department of Health Manage Center, Renji Hospital, School of Medicine, Shanghai Jiao Tong University, Shanghai, China

**Keywords:** subcortical vascular mild cognitive impairment, white matter hyperintensities, normal-appearing white matter, penumbra, diffusion tensor imaging

## Abstract

Loss of white matter (WM) integrity contributes to subcortical vascular mild cognitive impairment (svMCI). Diffusion tensor imaging (DTI) has revealed damage beyond the area of WM hyperintensity (WMH) including in normal-appearing WM (NAWM); however, the functional significance of this observation is unclear. To answer this question, in this study we investigated the relationship between microstructural changes in the WMH penumbra (WMH-P) and cognitive function in patients with svMCI by regional tract-based analysis. A total of 111 patients with svMCI and 72 patients with subcortical ischemic vascular disease (SIVD) without cognitive impairment (controls) underwent DTI and neuropsychological assessment. WMH burden was determined before computing mean values of fractional anisotropy (FA) and mean diffusivity (MD) within WMHs and WMH-Ps. Pearson’s partial correlations were used to assess the relationship between measurements showing significant intergroup differences and composite *Z*-scores representing global cognitive function. Multiple linear regression analysis was carried out to determine the best model for predicting composite *Z*-scores. We found that WMH burden in the genu, body, and splenium of the corpus callosum (GCC, BCC, and SCC respectively); bilateral anterior, superior, and posterior corona radiata; left sagittal stratum was significantly higher in the svMCI group than in the control group (*p* < 0.05). The WMH burden of the GCC, BCC, SCC, and bilateral anterior corona radiata was negatively correlated with composite *Z*-scores. Among diffusion parameters showing significant differences across the 10 WM regions, mean FA values of WMH and WMH-P of the BCC were correlated with composite *Z*-scores in svMCI patients. The results of the multiple linear regression analysis showed that the FA of WMH-P of the BCC and WMH burden of the SCC and GCC were independent predictors of composite *Z*-score, with the FA of WMH-P of the BCC making the largest contribution. These findings indicate that disruption of the CC microstructure—especially the WMH-P of the BCC—may contribute to the cognitive deficits associated with SIVD.

## Introduction

Age-related cognitive impairment (CI) is a significant public health concern that will become increasingly prevalent with the aging of the global population (Iadecola et al., 2019; Kaneshwaran et al., 2019). CI of vascular etiology is the second leading cause of cognitive deficits after Alzheimer’s disease (AD) worldwide, and may be the predominant cause in East Asia (Iadecola et al., 2019; Lam and Mok, 2019). Vascular cognitive impairment (VCI) is a syndrome that encompasses a wide spectrum of cognitive disorders associated with vascular disease (Gorelick et al., 2011, 2016; van der Flier et al., 2018). Subcortical VCI (SVCI), which is attributable to cerebral small vessel disease, accounts for approximately 50%–70% of VCI cases and ranges from subcortical vascular mild cognitive impairment (svMCI) to subcortical vascular dementia (SVaD; O’Brien et al., 2003; Lee et al., 2014; Shi and Wardlaw, 2016). The mechanisms of brain injury in SVCI include vessel occlusion, leakage of toxins, impaired vascular reactivity, decreased clearance of waste products, oligodendrocyte dysfunction, increased oxidation, and inflammation (Wardlaw et al., 2013). Treatment options for SVCI are limited and disease-modifying therapies are not yet available; reliable biomarkers for early diagnosis and disease monitoring are therefore urgently needed (Fu et al., 2019; Sang et al., 2020). A prodromal stage of SVaD has been recognized based on the observation that the progression from svMCI to SVaD can be prevented by managing risk factors and through drug treatments (Seo et al., 2010; Lee et al., 2014; Jung et al., 2018).

White matter hyperintensity (WMH) is the most common and critical magnetic resonance imaging (MRI) finding of SVCI (Moran et al., 2012; Wardlaw et al., 2013), and is caused by microvessel disruption, breakdown of the blood–brain barrier, small infarcts in white matter (WM), glia activation, loss of oligodendrocytes, and demyelination caused by chronic diffuse hypoperfusion or reduced cerebral blood flow (Pantoni, 2010; De Silva and Miller, 2016). The location and extent of WMH have been linked to CI (Papp et al., 2014; Altermatt et al., 2019; Lampe et al., 2019). In a previous study, tissue damage was observed to extend from WMHs to larger adjacent areas of normal-appearing WM (NAWM; Promjunyakul et al., 2016), suggesting an ischemic mechanism underlying WMH expansion. Furthermore, changes in NAWM were found to be associated with a decline in cognitive function as determined by diffusion tensor imaging (DTI; Huang et al., 2007; Papma et al., 2014; Hirsiger et al., 2017). NAWM surrounding WMHs that can only be detected on a microstructural level is referred to as the WMH-penumbra (WMH-P; Maillard et al., 2011); neuroimaging studies have revealed damage below the detection threshold in these non-lesioned WM areas (Promjunyakul et al., 2015, 2016; Wu et al., 2019). WMH does not fully explain the neuroimaging correlates of cognitive decline in SVCI, as histopathologic alterations in the WMH-P can also lead to CI (Simpson et al., 2007; Gouw et al., 2011; Promjunyakul et al., 2015); however, as these changes are subtle, it may be possible to prevent their aggravation through early intervention.

The pathology of SVCI involves structural abnormalities in WM that contribute to a disconnection syndrome and are correlated with loss of cognitive function (López-Gil et al., 2014; Tuladhar et al., 2016). Loss of WM tract integrity in SVCI has been observed by neuroimaging. For example, microstructural damage in the anterior corpus callosum (CC), internal and external capsules, and periventricular WM has been demonstrated in SVCI by tract-based spatial statistics (TBSS; Papma et al., 2014; Holland et al., 2015; Chen et al., 2018; Wang et al., 2020), which also revealed significant associations between microstructural changes in WM tracts underlying intra-and inter-hemispheric cerebral, thalamocortical, and cerebello-thalamic connections—including the CC and corona radiata—and cognitive performance (van der Holst et al., 2018; Mascalchi et al., 2019). An automated fiber quantification (AFQ) study found that changes in diffusion characteristics—especially in the right inferior fronto-occipital fasciculus and right inferior longitudinal fasciculus—may be involved in WMH-related MCI (Chen et al., 2020). However, AFQ can only analyze the central portion of WM fiber tracts, and cannot therefore exclude the contribution of other portions to CI; moreover, it does not allow tracing of all the fiber bundles in the human brain. TBSS can only be used to analyze voxels on the skeleton with the highest fractional anisotropy (FA), which also excludes a large part of the WM (Roine et al., 2015). Additionally, neither AFQ nor TBSS can be used to detect WMH or its penumbra in fibers.

In this study, we combined region-of-interest (ROI) and tract-based analyses to explore changes in WM microstructure in svMCI and their association with cognitive function. ROI and tract-based DTI analyses allow the parcellation of WM into pathways associated with specific functions, thereby allowing disease processes to be analyzed in terms of functional systems, which is an advantage over other methods (Liu et al., 2019; Chen et al., 2020). We aimed to characterize DTI changes in WMH and WMH-P of WM fiber bundles in order to explore the relationship between progressive damage to WM microstructure and decline in cognitive performance. We hypothesized that microstructural abnormalities reflected by WMH or NAWM (WMH-P) in specific WM fibers contribute to cognitive deficits in svMCI.

## Materials and Methods

### Participants

This research was approved by the Research Ethics Committee of Renji Hospital, School of Medicine, Shanghai Jiao Tong University. Signed, informed consent was obtained from each subject before their enrollment. All procedures were carried out in accordance with institutional guidelines.

SIVD patients (age range: 50–88 years) were recruited from the Neurology Department of Renji Hospital between January 2017 and December 2019. SIVD was defined as the presence of a subcortical WMH lesion with at least 1 lacunar infarct by neuroimaging (Erkinjuntti et al., 2000; Galluzzi et al., 2005). The patients were divided into two groups—svMCI (*n* = 111) and non-CI (control, *n* = 72)—according to previously published criteria (Petersen et al., 1999). The inclusion criteria were subjective cognitive complaints reported by the patient or caregiver; normal activities of daily living; quantifiable CI in 1 or more domains (memory, attention-executive function, language, or visuospatial function); and no dementia. The control group had neuropsychological test scores within the normal range and was matched to the svMCI group in terms of age, sex ratio, and education level. Exclusion criteria were as follows: dementia; neurodegenerative diseases (e.g., Parkinson’s disease or AD); severe brain atrophy, intracranial space-occupying lesions, microbleeds or hemorrhage revealed by susceptibility-weighted imaging; non-SVD-related WMH mimics (e.g., multiple sclerosis); psychiatric disease interfering with cognitive testing; alcoholic encephalopathy; recent or current use of certain drugs such as acetylcholine-esterase inhibitors, L-dopa, or neuroleptic agents; and MRI contraindications or known claustrophobia. Early SVCI is characterized by deficits in executive functioning or information processing speed with relatively intact retentive memory, and is less likely to produce subjective complaints; in contrast, AD or mixed CI are associated with memory problems. Thus, participants with memory complaints were excluded. All the patients were right-handed.

### Neuropsychological Assessment

All subjects underwent a battery of standardized neuropsychological evaluations carried out by an experienced neurologist (QX) within 1 week after the MRI scan. None of the subjects experienced transient ischemic attack or stroke in the interval between the MRI scan and neuropsychological testing. General cognitive function was evaluated with the Mini-Mental State Examination (MMSE) and Montreal Cognitive Assessment (MoCA). Additionally, the following tests were used to evaluate four specific cognitive domains as described in a previous study (Xu et al., 2014): (1) attention and executive function: Trail-Making Tests A and B, Stroop color-word test (Stroop C-T), and verbal fluency test; (2) visuospatial function: Rey-Osterrieth Complex Figure Test (copy); (3) language function: Boston Naming Test (30 items); and (4) memory function: auditory verbal learning test (short and long delayed free recall). The reference value (norm) used for each measurement was based on the mean score, which was determined from a small-scale pilot study conducted in Shanghai, China (Guo et al., 2007). Cognitive dysfunction was defined as −1.5 standard deviation (SD) relative to the normal value for at least 1 test item. To calculate performance on each cognitive domain, the raw score was transformed to a *Z*-score. Global cognitive function was represented by the composite *Z*-score, which was calculated as the average of *Z*-scores of all four cognitive domains.

### MRI Data Acquisition

Subjects were scanned using a 3.0T MRI scanner (Signa HDxt; GE HealthCare, Milwaukee, WI, USA) with an eight-channel phased array head coil. Head movement was restricted by placing foam padding around the head of the subject during the scan. Three-dimensional T1 high-resolution imaging, axial fluid-attenuated inversion recovery (FLAIR), and MR DTI scans were performed for each subject. Sagittal T1-weighted images covering the whole brain were acquired with a 3D-fast spoiled gradient recalled echo (SPGR) sequence with the following parameters: repetition time (TR) = 5.6 ms, echo time (TE) = 1.8 ms, inversion time (TI) = 450 ms, flip angle = 15°, matrix = 256 × 256, number of slices = 156, slice thickness = 1.0 mm, and field of view (FOV) = 256 × 256 mm^2^. The parameters used to obtain axial FLAIR images were as follows: TR = 9,075 ms, TE = 150 ms, TI = 2,250 ms, matrix = 256 × 256, number of slices = 66, slice thickness = 2 mm, and FOV = 256 × 256 mm^2^. DTI images were acquired using a spin-echo single shot echo-planar pulse sequence with the following parameters: TR = 17,000 ms, TE = 89.8 ms, matrix = 128 × 128, number of slices = 66, slice thickness = 2 mm, gap = 0, FOV = 256 × 256 mm^2^, number of excitations = 1, and gap = 0. Diffusion-sensitizing gradients were applied along 20 non-collinear directions with *a b* value of 1,000 s/mm^2^, a reference image with no diffusion gradients applied (b0 scan) was also acquird.

### MRI Data Processing

The T1 3D-SPGR images were uniformly segmented using Statistical Parametric Mapping 8^1^ running on MATLAB R2014a (MathWorks, Natick, MA, USA), from which tissue maps of gray matter (GM), WM and cerebrospinal fluid (CSF) were generated in the native space. WMH maps in the native space were segmented using T1 3D-SPGR and FLAIR images as raw materials with the lesion prediction algorithm in LST Toolbox v1.1.4^2^ (Schmidt et al., 2012). Brain volumes including total intracranial, GM, WM, CSF, and WMH volumes were calculated from T1 3D-SPGR images. Normalized WMH volume (NWMHV) was calculated as the WMH volume percentage relative to brain parenchyma volume, which was the sum of GM and WM volumes (NWMHV ‰ = measured WMH volume/brain parenchyma volume × 1,000‰).

WMH-P was defined as spatially peripheral WM tissue regions forming rings around WMH lesions and was assumed to have abnormal microstructure (de Groot et al., 2013; Nasrallah et al., 2019). To more precisely measure the WMH-P, WMH clusters were further subclassified into periventricular WMHs (PVWMH) and deep WMHs (DWMH) according to the so-called “continuity to ventricle rule” (Griffanti et al., 2018). Probabilistic maps for WMHs were processed by binarization. By linearly aligning the binary WMH map to the T1 3D-SPGR image, a NAWM layer mask for each dataset was created according to previous studies (Promjunyakul et al., 2015, 2016), which comprised 15 separate layers outside of PVWMHs and DWMHs. Each parallel layer successively dilated from the WMH by 1 mm.

DTI dataset was obtained using the PANDA v1.3.1 pipeline toolbox^3^, which is based on the FMRIB Software Library tools (Cui et al., 2013). The original DTI images were modified using the brain extraction tool and by eddy current-induced distortion and coregistration. FA and mean diffusivity (MD) were then simultaneously calculated with a mask created from the b0 image. FA and MD maps of each subject were first coregistered to the corresponding individual T1 3D-SPGR image using the b0 image and then smoothed with a 6 mm full width at half-maximum Gaussian kernel, then resliced into a 2 × 2 × 2 mm^3^ voxel-size matrix. According to the previous studies and our recent work (Promjunyakul et al., 2015, 2016; Wu et al., 2019), the size of FA and MD penumbras of PVWMH and DWMH were as follows: 6 mm for both FA and MD penumbras of PVWMH, 4 mm for the FA penumbra of DWMH, and 2 mm for the MD penumbra of DWMH. The FA and MD penumbras was gained by summing the FA or MD penumbra of PVWMH and DWMH.

**Figure 1:**
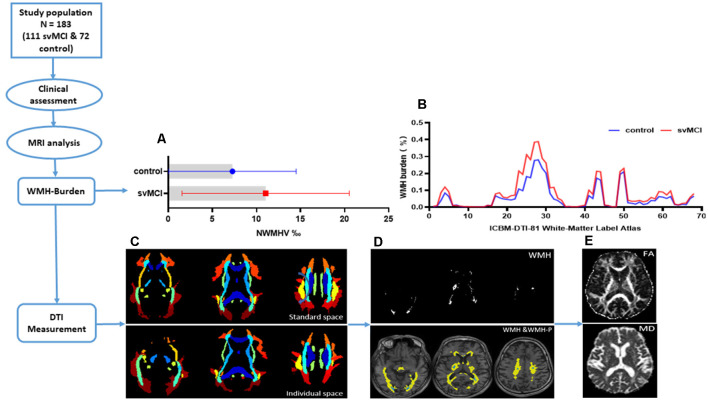
The flowchart showing data processing pipeline and results from some of the intermediate steps. **(A)** Bars showing mean and range of variation of normal-appearing white matter hyperintensity volume (NWMHV) for subcortical vascular mild cognitive impairment (svMCI) and control group. The red bar and small square stood for svMCI group while the blue bar and small circle stood for control group. **(B)** White matter hyperintensity (WMH) burden in each WM fiber tract based on the ICBM-DTI-81 WM label atlas. The atlas we used was a 60% probability map which included 68 WM fiber tracts in total. The red line corresponded to svMCI group and the blue line corresponded to control group. **(C)** ICBM-DTI-81 WM label atlas in standard (MNI) space and in individual space of one subject. **(D)** The WMH and WMH penumbra (WMH-P) mask image of the same subject. The white area represented for the general WMH lesion. The blue area represented for periventricular WMH (PVWMH) and deep WMH (DWMH), and the yellow area represented for penumbras surrounding either PVWMH or DWMH. **(E)** The fractional anisotropy (FA) and mean diffusivity (MD) map of the same subject.

We used the Johns Hopkins University Inventory of Cognitive Bias in Medicine (ICBM)-DTI-81 WM label atlas (Mori et al., 2008) as an anatomic guide to parcel the WM into 68 ROI, with each ROI representing a labeled region in the atlas. We inversely coregistered the ICBM-DTI-81 WM atlas to individual space, then calculated the WMH burden in each of the 68 WM tracts for each subject. Taking into consideration differences in brain volume across subjects, WMH burden was defined and measured as the percentage of WMH voxels in all single WM fiber bundle voxels. Thus, for each subject, all relevant images—i.e., diffusion as well as brain segmentation maps, including WMH and WMH-P maps and subject-specific WM ROI mask—were uniformly co-aligned in the individual T1 3D-SPGR space. The mean values of FA and MD in WMH and WMH-P of WM ROIs were extracted. Figure 1 depicts the data processing pipeline and results from some of the intermediate steps.

### Statistical Analysis

All statistical analyses were performed using SPSS v25 (IBM, Armonk, NY, USA). The Kolmogorov–Smirnov test was used to determine whether the data were normally distributed. The independent sample *t*-test was used to compare continuous variables and the χ_2_-test was used for all other variables. False discovery rate corrections were applied (*q*-value = 0.05). Partial correlations were used to evaluate correlations between WMH burden, DTI parameters, and composite *Z*-scores. A stepwise multiple linear regression model was used to predict the independent effect of each variable on composite *Z*-score. Variables obtained in the two steps that were strongly correlated with composite *Z*-score were used as predictors. Collinearity was tested using the variance inflation factor (VIF); variables with VIFs ≥5 were removed because of multicollinearity. Durbin–Watson statistics were used to detect the presence of autocorrelation. All *p*-value <0.05 were considered statistically significant.

## Results

### Demographic and Clinical Characteristics of the Study Population

There were no significant differences in age, sex ratio, or education level between svMCI and non-CI (control) groups. NWMHV was significantly higher (Figure 1A) whereas composite *Z*-scores and MMSE and MoCA scores were lower (Table 1) in svMCI patients than in control subjects.

**Table 1:** Demographic, imaging and clinical characteristics of patients with SIVD.

	svMCI group (*n* = 111)	Control group (*n* = 72)	*p*
Demographic characteristics
Age (years)	65.2 ± 7.8	65.4 ± 7.6	0.92
Sex ratio [Female, N (%)]	27 (24.3)	11 (15.3)	0.14
Edcation level (years)	10.4 ± 2.9	11.2 ± 3.2	0.06
Imaging characteristics
NWMHV, ‰	1.10 ± 0.95	0.73 ± 0.72	0.005**
Global cognitive scores
MMSE	27.3 ± 2.1	28.6 ± 1.37	<0.001***
MoCA	22.2 ± 3.3	25.9 ± 2.4	<0.001***
Composite *Z*-score	−0.84 ± 0.85	0.17 ± 0.50	<0.001***

Data represent number (percentage), mean ± standard deviation (SD); two-sample *t-tests* were performed to assess group comparison for age, education level, NWMHV, MMSE, MoCA, composite *Z*-score, and χ_2_-test for sex ratio. *P*-value <0.05 was considered to be statistically significant. NWMHV (normalized WMH volumes, ‰), measured WMH volume/brain-parenchymal volume × 1,000‰; MMSE, Mini Mental State Examination; MoCA, Montreal Cognitive Assessment; svMCI, subcortical vascular mild cognitive impairment. **p < 0.01, ***p < 0.001.

### WMH Burden and DTI Parameters

**Figure 2:**
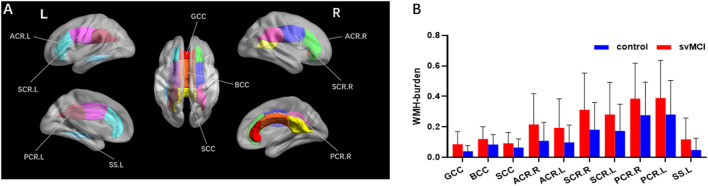
Results of group comparison of WMH burden. Both the visual map **(A)** and the column diagram **(B)** showed 10 WM fiber tracts altogether that had significant difference of WMH burden between svMCI and control groups. **(B)** The red column corresponded to svMCI group and the blue column corresponded to control group. GCC, genu of corpus callosum; BCC, body of corpus callosum; SCC, splenium of corpus callosum; ACR.R, right anterior corona radiata; ACR.L, left anterior corona radiata; SCR.R, right superior corona radiata; SCR.L, left superior corona radiata; PCR.R, right posterior corona radiata; PCR.L, left posterior corona radiata; SS.L, left sagittal stratum.

**Figure 3:**
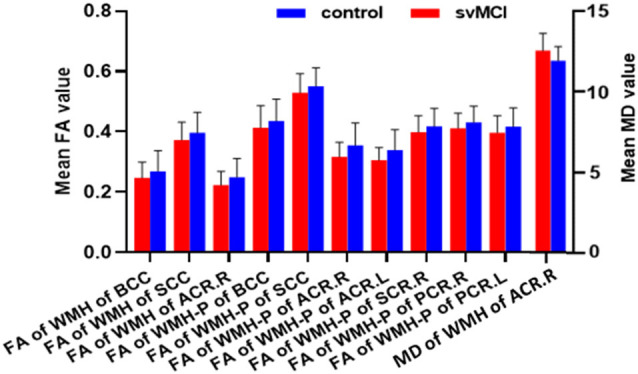
Results of group comparison of diffusion tensor imaging (DTI) parameters. Column diagram showing diffusion parameters based on the 10 WM fiber tracts which had significant between-group difference of WMH burden. The red column corresponded to svMCI group and the blue column corresponded to control group. The left *Y*-axis indicated mean FA value and the right *Y*-axis indicated mean MD value.

The svMCI group had higher WMH burden compared to control subjects in 10 fibers: the genu, body, and splenium of the CC (GCC, BCC, and SCC, respectively); bilateral anterior, superior, and posterior corona radiata (ACR, SCR, and PCR, respectively); and left sagittal stratum (all *q* < 0.05; Figure 2, Table 2). For the 10 WM fibers, 11 DTI parameters were found to differ significantly between groups including FA of WMH in the BCC, SCC, and right ACR; MD of WMH of right ACR; FA of WMH-P of BCC, SCC, bilateral ACR, bilateral PCR and right SCR (*p* < 0.05, Figure 3, Table 3). The FA values of WMH and WMH-P were lower while MD value was higher in the svMCI group compared to the control group.

**Table 2:** White matter hyperintensity (WMH) burdens of WM fiber bundles that had significant differences between svMCI and control group.

WM regions	svMCI mean ± SD	Control mean ± SD	*q*
GCC	0.086 ± 0.084	0.040 ± 0.038	0.001
BCC	0.120 ± 0.081	0.084 ± 0.065	0.019
SCC	0.092 ± 0.072	0.064 ± 0.057	0.041
ACR.R	0.215 ± 0.203	0.108 ± 0.122	0.002
ACR.L	0.194 ± 0.190	0.098 ± 0.114	0.002
SCR.R	0.312 ± 0.240	0.182 ± 0.179	0.002
SCR.L	0.281 ± 0.212	0.174 ± 0.175	0.006
PCR.R	0.385 ± 0.233	0.277 ± 0.217	0.018
PCR.L	0.389 ± 0.247	0.280 ± 0.225	0.023
SS.L	0.118 ± 0.140	0.048 ± 0.077	0.002

*Data represent mean ± standard deviation (SD). *q*-value referred to false discovery rate-corrected *p*-value. svMCI, subcortical vascular mild cognitive impairment; GCC, genu of corpus callosum; BCC, body of corpus callosum; SCC, splenium of corpus callosum; ACR.R, right anterior corona radiata; ACR.L, left anterior corona radiata; SCR.R, right superior corona radiata; SCR.L, left superior corona radiata; PCR.R, right posterior corona radiata; PCR.L, left posterior corona radiata; SS.L, left sagittal stratum*.

**Table 3:** Diffusion tensor imaging (DTI) parameter values of the WMH and WMH-P of WM fiber bundles that had significant WMH-burden differences between svMCI and control group.

		svMCI mean ± SD	Control mean ± SD	*p*
GCC	FA of WMH	0.18 ± 0.06	0.20 ± 0.11	0.380
	MD of WMH	15.98 ± 2.46	16.79 ± 3.30	0.119
	FA of WMH-P	0.342 ± 0.07	0.345 ± 0.08	0.843
	MD of WMH-P	15.20 ± 2.21	15.54 ± 2.26	0.318
BCC	FA of WMH	0.25 ± 0.05	0.27 ± 0.07	0.021*
	MD of WMH	14.03 ± 1.93	13.61 ± 1.55	0.143
	FA of WMH-P	0.41 ± 0.07	0.44 ± 0.07	0.046*
	MD of WMH-P	14.87 ± 2.19	14.39 ± 1.92	0.129
SCC	FA of WMH	0.37 ± 0.06	0.40 ± 0.07	0.017*
	MD of WMH	13.08 ± 1.93	12.99 ± 1.54	0.730
	FA of WMH-P	0.53 ± 0.06	0.55 ± 0.06	0.032*
	MD of WMH-P	14.73 ± 1.84	14.39 ± 1.45	0.186
ACR.R	FA of WMH	0.22 ± 0.05	0.25 ± 0.06	0.010*
	MD of WMH	12.57 ± 1.09	11.94 ± 0.88	0.006**
	FA of WMH-P	0.32 ± 0.05	0.35 ± 0.07	<0.001***
	MD of WMH-P	11.27 ± 0.82	11.21 ± 0.82	0.620
ACR.L	FA of WMH	0.22 ± 0.04	0.24 ± 0.05	0.117
	MD of WMH	12.54 ± 1.18	12.25 ± 1.17	0.243
	FA of WMH-P	0.31 ± 0.04	0.34 ± 0.07	<0.001***
	MD of WMH-P	11.38 ± 0.90	11.20 ± 0.60	0.185
SCR.R	FA of WMH	0.31 ± 0.08	0.32 ± 0.08	0.357
	MD of WMH	11.92 ± 1.09	11.90 ± 1.70	0.921
	FA of WMH-P	0.40 ± 0.06	0.42 ± 0.06	0.026*
	MD of WMH-P	11.27 ± 1.25	11.08 ± 1.74	0.519
SCR.L	FA of WMH	0.314 ± 0.08	0.315 ± 0.08	0.955
	MD of WMH	11.87 ± 1.10	11.76 ± 1.10	0.610
	FA of WMH-P	0.40 ± 0.05	0.41 ± 0.07	0.139
	MD of WMH-P	11.18 ± 1.16	11.05 ± 0.66	0.500
PCR.R	FA of WMH	0.30 ± 0.07	0.32 ± 0.06	0.058
	MD of WMH	12.07 ± 1.90	11.97 ± 1.37	0.742
	FA of WMH-P	0.41 ± 0.05	0.43 ± 0.05	0.016*
	MD of WMH-P	11.52 ± 2.02	11.63 ± 2.16	0.746
PCR.L	FA of WMH	0.29 ± 0.08	0.30 ± 0.08	0.555
	MD of WMH	12.00 ± 1.80	11.67 ± 1.25	0.267
	FA of WMH-P	0.40 ± 0.06	0.42 ± 0.06	0.024*
	MD of WMH-P	11.18 ± 1.51	10.99 ± 1.25	0.446
SS.L	FA of WMH	0.30 ± 0.05	0.31 ± 0.05	0.152
	MD of WMH	14.82 ± 2.04	15.21 ± 2.86	0.454
	FA of WMH-P	0.41 ± 0.05	0.42 ± 0.05	0.070
	MD of WMH-P	12.49 ± 1.18	12.57 ± 1.56	0.725

Data represent mean ± standard deviation (SD). *P*-value <0.05 was considered to be statistically significant. WMH, white matter hyperintensity; WMH-P, white matter hyperintensity penumbra; FA, fractional anisotropy; MD, mean diffusivity (MD values are not marked in unit since being post-processed); svMCI, subcortical vascular mild cognitive impairment; GCC, genu of corpus callosum; BCC, body of corpus callosum; SCC, splenium of corpus callosum; ACR.R, right anterior corona radiata; ACR.L, left anterior corona radiata; SCR.R, right superior corona radiata; SCR.L, left superior corona radiata; PCR.R, right posterior corona radiata; PCR.L, left posterior corona radiata; SS.L, left sagittal stratum. *p < 0.05, **p < 0.01, ***p < 0.001.

### Associations Between Influential Factors and General Cognitive Function

**Figure 4:**
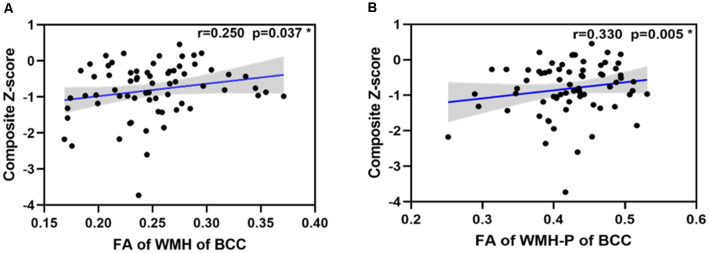
Scatterplot of the partial correlations of composite *Z*-score with DTI parameters on WM tracts **(A,B)**. For panels **(A,B)**, lines and shaded areas represented the correlation coefficient and each 95% confidence interval (CI), respectively.

In svMCI patients, the WMH burden of the GCC, BCC, SCC, bilateral ACR, and SCR was negatively correlated with composite *Z*-scores (Table 4), with age, sex ratio, and education as covariates. In the svMCI group, the FA values of WMH and WMH-P in the BCC were positively correlated with composite *Z*-scores (Figure 4, Table 5), with age, sex ratio, education level, and corresponding WMH burden as covariates. In the control group, only the FA value of WMH in the BCC had significantly negative correlation with composite *Z*-score (Table 6), with age, sex ratio and education level as covariates. A regression equation was developed based on the relationship between observed and predicted values for composite *Z*-scores. The composite *Z*-score was significantly and independently associated with FA of WMH-P in the BCC, WMH burden of the SCC, and WMH burden of the GCC, with the FA of WMH-P in the BCC making the most significant contribution (Table 7).

**Table 4:** Partial correlations between WMH burden and composite *Z*-scores in svMCI group.

WMH burden	Composite *Z*-score	
	*r*	*p*
GCC	−0.321	0.006**
BCC	−0.262	0.027*
SCC	−0.369	0.002**
ACR.R	−0.303	0.010*
ACR.L	−0.284	0.016*
SCR.R	−0.347	0.003**
SCR.L	−0.296	0.012*
PCR.R	−0.218	0.074
PCR.L	−0.181	0.139
SS.L	−0.083	0.503

P-value <0.05 was considered to be statistically significant. GCC, genu of corpus callosum; BCC, body of corpus callosum; SCC, splenium of corpus callosum; ACR.R, right anterior corona radiata; ACR.L, left anterior corona radiata; SCR.R, right superior corona radiata; SCR.L, left superior corona radiata; PCR.R, right posterior corona radiata; PCR.L, left posterior corona radiata; SS.L, left sagittal stratum. *p < 0.05, **p < 0.01.

**Table 5:** Partial correlations between DTI parameters of WMH and WMH-P of significant WM fiber bundles and composite *Z*-scores in svMCI group adjusted for age, sex ratio, education level, and WMH burden.

DTI parameter	Composite *Z*-score	
	*r*	*p*
FA of WMH of BCC	0.25	0.037*
FA of WMH of SCC	−0.073	0.555
FA of WMH of ACR.R	−0.056	0.693
MD of WMH of ACR.R	−0.03	0.841
FA of WMH-P of BCC	0.33	0.005**
FA of WMH-P of SCC	0.219	0.075
FA of WMH-P of ACR.R	−0.007	0.955
FA of WMH-P of ACR.L	0.025	0.844
FA of WMH-P of SCR.R	0.109	0.378
FA of WMH-P of PCR.R	0.059	0.635
FA of WMH-P of PCR.L	0.061	0.625

P-value <0.05 was considered to be statistically significant. BCC, body of corpus callosum; SCC, splenium of corpus callosum; ACR.R, right anterior corona radiata; ACR.L, left anterior corona radiata; SCR.R, right superior corona radiata; PCR.R, right posterior corona radiata; PCR.L, left posterior corona radiata; WMH, white matter hyperintensity; WMH-P, white matter hyperintensity penumbra. *p < 0.05, **p < 0.01.

**Table 6:** Partial correlations between DTI parameters of WMH and WMH-P in significant WM fiber bundles and composite *Z*-scores in control group adjusted for age, sex ratio and education level.

DTI parameter	Composite *Z*-score	
	*r*	*p*
FA of WMH of BCC	−0.530	0.011*
FA of WMH of SCC	−0.165	0.464
FA of WMH of ACR.R	−0.055	0.806
MD of WMH of ACR.R	0.197	0.380
FA of WMH-P of BCC	−0.393	0.070
FA of WMH-P of SCC	0.107	0.636
FA of WMH-P of ACR.R	0.088	0.696
FA of WMH-P of ACR.L	0.147	0.515
FA of WMH-P of SCR.R	0.089	0.693
FA of WMH-P of PCR.R	0.123	0.587
FA of WMH-P of PCR.L	0.196	0.383

P-value <0.05 was considered to be statistically significant. BCC, body of corpus callosum; SCC, splenium of corpus callosum; ACR.R, right anterior corona radiata; ACR.L, left anterior corona radiata; SCR.R, right superior corona radiata; PCR.R, right posterior corona radiata; PCR.L, left posterior corona radiata; WMH, white matter hyperintensity; WMH-P, white matter hyperintensity penumbra. *p < 0.05.

**Table 7:** Stepwise multiple linear regression of composite *Z*-scores.

	*R*	*R*^2^ (adjusted)	*β*	*t*	*p*	VIF	Durbin–Watson
(Constant)	−	−	−	−2.456	0.017	−	2.094
FA of WMH-P of BCC	0.562	0.295	0.432	4.071		<0.001	1.134
WMH burden of SCC	0.493	0.232	−0.236	−2.334	0.023	1.029	
WMH burden of GCC	0.596	0.325	−0.214	−2.004	0.049	1.154	

*P-value <0.05 was considered to be statistically significant. BCC, body of corpus callosum; SCC, splenium of corpus callosum; GCC, genu of corpus callosum; VIF, variance inflation factor; WMH, white matter hyperintensity; WMH-P, white matter hyperintensity penumbra*.

## Discussion

The main objective of this study was to investigate the relationship between microstructural abnormalities in WMH and NAWM (WMH-P) in specific fiber tracts and the development of CI in svMCI, which is an early stage of SVCI that has been overlooked in most previous work. Thus, our results provide prospective information that can guide early therapeutic decision-making.

We found that the WMH burden in some parts of the CC and corona radiata was significantly higher in patients with svMCI than in control subjects. FA values of WMH in the BCC, SCC, and right ACR and those of WMH-P in the BCC, SCC, bilateral ACR, right SCR, and bilateral PCR were lower whereas the MD value of WMH in the right ACR was higher in svMCI patients. Furthermore, a significant correlation between FA of WMH-P in the BCC and general cognitive function was found in svMCI patients but not in control subjects.

Consistent with previous studies (Lin et al., 2015; Tu et al., 2017; Chen et al., 2018; Liu et al., 2019; Wang et al., 2020), the strongest associations between abnormal DTI parameters and cognitive decline were found in the CC and corona radiata. As one of the most extensively myelinated brain regions, the CC is composed of fibers arising from large pyramidal neurons, with the anterior portion containing axons of corticocortical communicating fibers from corresponding posterior lobar regions (Matsunami et al., 1994; Abe et al., 2004). Fibers from these regions converge in the corona radiata, which contains projection fibers that are involved in information transmission. The critical roles of these ascending/descending pathways and inter-hemispheric connections in cognitive function can explain the CI associated with microvascular damage in the CC and corona radiata. WM tracts in the CC harboring commissural fibers connecting the frontal lobe and other cortical areas were found to be damaged at the early stage of SVCI (Reginold et al., 2016; Moscufo et al., 2018), indicating that SVCI is associated with the loss of integrity of the CC microstructure. The association between WM tract abnormalities and CI was also evidenced by the fact that SVCI lesions were present not only in certain areas but along the entire fiber structure.

The correlation between FA of WMH in the BCC and cognitive function was positive in svMCI patients and negative in control subjects. This discrepancy may be explained by the heterogeneity of pathologic changes that give rise to WMHs, which include incomplete ischemia mainly related to cerebral small vessel arteriolosclerosis; disruption of the blood-brain barrier; vasomotor reactivity, autoregulation, and endothelial dysfunction; systemic oxidative stress; inflammation; and chronic edema (Bakker et al., 1999; Pantoni, 2002; Hassan et al., 2003; Chung and Hu, 2010; Xu et al., 2010), which may coexist with tissue damage and repair. SIVD has a relatively long disease course. Although WMH progression was traditionally thought to be continuousand uniform, it is now known to be a dynamic and highly variable process that sometimes regresses (Schmidt et al., 2002; van Leijsen et al., 2017). The attenuation of WMHs is often incomplete because repair processes can be disrupted by the inhibitory responses of glial cells (Hayakawa and Lo, 2016). Thus, changes in FA values are complex and can be difficult to interpret.

Previous studies have shown that ischemic changes in the WMH-P reflect those in WMHs, with the only difference being that the former are more subtle and prodromal (Maillard et al., 2011; Jiaerken et al., 2019). We found a significant association between FA of WMH-P in the BCC and composite *Z*-scores in svMCI subjects but not in control subjects, underscoring the contribution of the penumbra to the cognitive deficits observed in svMCI, although the mechanistic details remain unclear.

We found that diffusion features of the WMH-P distinguished svMCI patients from control subjects, indicating that the degree of abnormality in WMH-P microstructure plays an important role in cognitive decline, possibly through a decreased connectivity caused by demyelination and axonal damage (Moscufo et al., 2018). The WMH-P represents a region of milder injury surrounding WMH lesions and has a higher probability of progressing to WMH than NAWM outside the penumbra (Maillard et al., 2011); moreover, while it is not in itself destructive, the WMH-P is a risk factor for future brain injury that presumably contributes to CI. The histopathologic origins of age-related WM degeneration include gliosis, degeneration in myelinated axons, and importantly, small vessel changes whose direct effects on WM integrity may induce the transition of WMH-P to WMH. Hypertension, diabetes, and other SVCI risk factors at their earliest stages are accompanied by progressive and subtle WM degeneration that manifests as WMH (de Leeuw et al., 2002; van Dijk et al., 2004). This is relevant to the clinical interpretation of WMH-P, which is considered as a more diffuse and incipient brain injury, and may represent a novel treatment target that, if salvaged, might modify the time course of progressive WMH and its cognitive consequences. Additionally, early treatment of reversible cardiovascular risk factors may slow the progression of WM damage in SVCI. Previous longitudinal studies found that some WMHs regress after minor stroke, with potential improvement of neurologic outcomes. Thus, WMH is reversible, which may be attributable to the transience of blood-brain barrier disruption and resultant changes in interstitial fluid (Cho et al., 2015; Ding et al., 2015; Wardlaw et al., 2017).

We also found that the MD of WMH in the right ACR was higher in svMCI patients than in control subjects, which was the only positive result for MD value. This suggests that MD is less sensitive than FA in detecting the degree of WM damage—in disagreement with previous studies on AD (Jin et al., 2017) or CSVD (Chen et al., 2020)—and that the magnitude of diffusion is a more sensitive index than the direction, possibly because microstructural changes in the chronic stage of injury reflects a combination of quantitative and directional injury caused by Wallerian degeneration.

There were several limitations to our study. First, the study design was cross-sectional and the sample size was small; longitudinal studies with a larger sample are needed in the future. Second, as with all MRI studies of svMCI we were limited by the lack of pathologically confirmed cases, although we used both neuropsychological assessment and MRI for diagnosis. Third, we excluded patients with serious brain atrophy and microbleeds but did not control for the number of lacunar infarcts, which may have confounded our analysis of the association between WMH and its penumbra and cognitive performance. In our future work, we will quantify and model this confounder to clarify the role of the WMH-P in cognitive function. Fourth, the cognitive data were combined into a composite *Z*-score; however, analyses of individual cognitive domains are warranted as they involve different brain regions. Finally, the field strength and resolution of the MRI could be improved by including a larger number of gradient directions for a more sensitive measure of changes in WM microstructure.

## Conclusions

The significant fiber damage observed in svMCI suggests that loss of microstructural integrity of specific WM fibers—especially those of WMH-P in the BCC—contributes to cognitive deficits in patients with svMCI. These findings can aid the early diagnosis of svMCI so that timely and appropriate interventions can be initiated to slow cognitive decline in patients and reduce the progression from svMCI to SVaD.

## Data Availability Statement

The data analyzed in this study is subject to the following licenses/restrictions: de-identified data are available from the corresponding author upon reasonable request subject to a material transfer agreement. Requests to access these datasets should be directed to YQ, jacobqiu1@163.com.

## Ethics Statement

The studies involving human participants were reviewed and approved by Research Ethics Committee of Renji Hospital, School of Medicine, Shanghai Jiao Tong University. The patients/participants provided their written informed consent to participate in this study.

## Author Contributions

YZ and YS designed the study, provided the resources, wrote, reviewed, and edited the manuscript. YQ, LY, and XG contributed to the methodology, wrote the original draft, and curated data. YQ, YW, and XW processed the data. YZ, QX, and JX reviewed the draft and manuscript. All authors contributed to the article and approved the submitted version.

## Conflict of Interest

The authors declare that the research was conducted in the absence of any commercial or financial relationships that could be construed as a potential conflict of interest.

## Footnotes

^1^http://www.fil.ion.ucl.ac.uk/spm/software/spm8/

^2^www.statistical-modeling.de/lst.html

^3^http://www.nitrc.org/projects/panda
